# Extracellular vesicles from A23187-treated neutrophils cause cGAS-STING-dependent IL-6 production by macrophages

**DOI:** 10.3389/fimmu.2022.949451

**Published:** 2022-07-29

**Authors:** Edwina R. Allen, Kaitlyn M. Whitefoot-Keliin, Ellen M. Palmatier, Andrew R. Mahon, Mallary C. Greenlee-Wacker

**Affiliations:** Department of Biology, Central Michigan University, Mount Pleasant, MI, United States

**Keywords:** EVs, exosomes, microvesicles, cGAS, STING, inflammation, ectosomes

## Abstract

In response to several types of bacteria, as well as pharmacological agents, neutrophils produce extracellular vesicles (EVs) and release DNA in the form of neutrophil extracellular traps (NETs). However, it is unknown whether these two neutrophil products cooperate to modulate inflammation. Consistent with vital NETosis, neutrophils challenged with *S. aureus*, as well as those treated with A23187, released significantly more DNA relative to untreated or fMLF-treated neutrophils, with no lysis occurring for any condition. To test the hypothesis that EVs generated during NETosis caused macrophage inflammation, we isolated and characterized EVs from A23187-treated neutrophils (A23187-EVs). A23187-EVs associated with neutrophil granule proteins, histone H3, transcription factor A, mitochondrial (TFAM), and nuclear and mitochondrial DNA (mtDNA). We showed that DNA from A23187-EVs, when transfected into macrophages, led to production of IL-6 and IFN-α2, and this response was blunted by pre-treatment with the STING inhibitor H151. Next, we confirmed that A23187-EVs were engulfed by macrophages, and showed that they induced cGAS-STING-dependent IL-6 production. In contrast, neither EVs from untreated or fMLF-treated cells exhibited pro-inflammatory activity. Although detergent-mediated lysis of A23187-EVs diminished IL-6 production, removal of surface-associated DNA with DNase I treatment had no effect, and A23187-EVs did not induce IFN-α2 production. Given these unexpected results, we investigated whether macrophage mtDNA activated the cGAS-STING signaling axis. Consistent with mitochondrial outer membrane permeabilization (MOMP), a defined mechanism of mtDNA release, we observed macrophage mitochondrial membrane depolarization, a decrease in cytosolic Bax, and a decrease in mitochondrial cytochrome c, suggesting that macrophage mtDNA may initiate this EV-dependent signaling cascade. All together, these data demonstrate that A23187-EVs behave differently than transfected NET- or EV-DNA, and that neutrophil-derived EVs could be used as a model to study NF-κB-dependent STING activation.

## Introduction

Neutrophils are the most abundant leukocyte in blood, and neutrophil-derived EVs are elevated during sepsis and other inflammatory conditions (1–4). EVs are nano-sized, membrane-bound particles involved in intercellular communication (5). Although an anti-inflammatory function was originally prescribed to neutrophil-derived EVs (6, 7), a vast number of studies have subsequently uncovered immunomodulatory and pro-inflammatory activities. Bacteria and pharmacological agents can result in the generation of EVs with wide-ranging activities on recipient cells, but the pro-inflammatory effects are driven by activation of pattern recognition receptors (4). For example, compared to EVs isolated from neutrophils treated with either fMLF or PMA, *M. tuberculosis*-treated neutrophils produce EVs that induce TLR2/6-dependent pro-inflammatory cytokine production by macrophages, as well as increase co-stimulatory molecule expression, and increase bacterial killing by macrophages (8). Whether EVs released during sterile inflammation can activate pattern recognition receptors is an active area of investigation.

Similar to EVs, NETs are released from neutrophils in response to bacteria and select pharmacological agents, and are elevated in inflammatory conditions (9). NETs are released during NETosis and are comprised of decondensed DNA associated with neutrophil granule and DNA-binding proteins (10, 11). NETs can ensnare bacteria, but are also associated with adverse outcomes for the host, including causing thrombosis, autoantibody generation, and tissue and organ damage (12–14). Despite these findings, *in vitro* data showing NETs are pro-inflammatory are limited. Co-culturing NETs with macrophages results in a greater anti bacterial response (15), priming for inflammasome activation (16), and induction of programmed cell death (17–19). However, NETs enter human macrophages silently and do not trigger NF-κB activation (as measured by TNF-α and IL-6 production), inflammasome activation, or type I interferon production (20). Intracellular dsDNA can trigger several DNA pattern recognition pathways, including cGAS-STING activation. In this pathway, cytosolic DNA serves as a catalyst for dimerization of cyclic GMP-AMP Synthase (cGAS), leading to 2’3’-cGAMP production, dimerization of Stimulator of Interferon Genes (STING), and subsequent activation of transcription factors IRF-3 and NF-κB (21). Accordingly, transfection of NET DNA into macrophages activates the cGAS-STING signaling axis (22), suggesting that the mechanism of NET uptake changes the macrophage response.

EVs and NETs can be produced at the same time, and in prior work using EVs from human neutrophils challenged with *S. taphylococcus aureus*, a bacteria which causes NET production, we showed that mitochondrial, nuclear, and bacterial DNA associated electrostatically with EVs (23). Likewise, murine bone marrow neutrophils stimulated with PMA released NET DNA, and DNA bound to neutrophil-derived EVs (12). Although NETs and EVs interact, little is known of the functional consequences on macrophage activation and pro-inflammatory cytokine production, and whether EVs generated during NETosis are pro-inflammatory.

A23187 is a calcium ionophore, and induces a rapid, NOX-independent, non-lytic form of NETosis (vital NETosis) in neutrophils (24, 25). EVs from neutrophils treated with A23187 (A23187-EVs) induce damage to vascular endothelial cells in the presence of H_2_O_2_ (26), but their action on macrophages has not been investigated. Here, we isolated and characterized EVs from human neutrophils treated with A23187. Compared to spontaneously generated EVs or fMLF-EVs, A23187-EVs associated with DNA and possessed elevated levels of myeloperoxidase (MPO), neutrophil elastase (NE), TFAM, and histone H3. To test the hypothesis that A23187-EVs activate pattern recognition receptors and drive a pro-inflammatory response, we measured macrophage pro-inflammatory cytokine production following stimulation with these different EV subsets. Our results show that A23187-EVs activate the cGAS-STING signaling pathway. However, we observed no role for surface EV-DNA in triggering this inflammation. Instead, consistent with a model of cisplatin-induced acute kidney injury (27), we observed no type I interferon production, and increased mitochondrial dysfunction following treatment with A23187-EVs, suggesting that in response to A23187-EVs, macrophage mtDNA may activate the cGAS-STING signaling axis.

## Materials and methods

### Reagents and antibodies

All reagents, including A23187, N-Formylmethionyl-leucyl-phenylalanine (fMLF), and phorbol 12-myristate 13-acetate (PMA), were purchased from Sigma-Aldrich (St. Louis, MO) unless otherwise indicated. Triton X-100 was purchased from Fisher Scientific (Waltham, MA). Heparin, Pierce lactate dehydrogenase (LDH) cytotoxicity assay kit, Pierce BCA kit, Whatman Puradisc 0.2 µm polyethersulfone filters, and Halt protease inhibitor cocktail were purchased from Thermo Fisher Scientific (Waltham, MA). Sytox Green, sulfur/aldehyde latex beads, Quant-iT PicoGreen dsDNA assay kit, MitoProbe JC-1 assay, and antibodies against neutrophil elastase, and GRP94 were purchased from Invitrogen (Waltham, MA). ELISA kits for IL-6 and IFN-α2, and antibodies against CD63-PE, CD14-APC, myeloperoxidase (MPO), TFAM, and GAPDH were purchased from BioLegend (San Diego, CA). H151, g140, and double-stranded *E. coli* DNA were purchased from *Invivo*gen (San Diego, CA). Mini-PROTEAN TGX pre-cast stain free gels and QX200 ddPCR EvaGreen Supermix were purchased from Bio-Rad (Hercules, CA). Staurosporine, poly(I:C), and antibodies against TOM20 and Bax were purchased from Cell Signaling Technologies (Danvers, MA). AffiniPure goat-anti-mouse-HRP and AffiniPure goat-anti-rabbit-HRP were purchased from Jackson ImmunoResearch (West Grove, PA). Anti-flotillin-1 and anti-citrullinated histone H3 were purchased from Abcam (Waltham, MA). For cell isolation and cell culture, the following reagents were used: dextran (Pharmacosmos, Holbaek, Denmark), Ficoll-Paque PLUS (VWR, Atlanta, GA), sterile 0.9% saline, cell culture grade water, Lonza RPMI 1640 and RPMI 1640 without phenol red, and 0.25% trypsin-EDTA (Fisher Scientific, Waltham, MA). Uranyl acetate (Polysciences Inc, Warrington, PA), formvar/carbon-coated copper grids, paraformaldehyde and glutaraldehyde (Electron Microscopy Sciences, Hatfield, PA) were purchased for transmission electron microscopy (TEM). GelRed Nucleic Acid Gel Stain (Biotium, Fremont, CA), DNeasy blood and tissue kit (Qiagen), and recombinant DNase I (Takara) were purchased to analyze DNA.

### *Staphylococcus aureus* culture

The USA300 LAC strain of *S.aureus* was an in-kind gift from Dr. F. R. DeLeo (Rocky Mountain Laboratories, NIAID). *S. aureus* was cultured in tryptic soy broth overnight at 37°C, with agitation at 180 rpm. Bacteria were diluted to an OD_550_ of 0.05 and grown to mid-logarithmic phase for 3 hours. Bacteria were then pelleted and resuspended in 20 mM HEPES in HBSS with divalent cations, then opsonized in 10% pooled human serum at 37°C while tumbling for 20 minutes. Following this incubation, bacteria were washed twice at 2800 × g for 5 minutes at 4°C using RPMI 1640 without phenol red.

### Isolation of neutrophils and macrophages

Venous blood was collected from consented healthy donors with approval by the Institutional Review Board at Central Michigan University (Mount Pleasant, MI, USA). Neutrophils and macrophages were isolated as described in prior work (28). Briefly, heparinized blood was subject to dextran sedimentation, followed by density gradient separation with Ficoll Paque PLUS. After centrifugation, erythrocytes sedimented in the bottom layer with neutrophils were removed by hypotonic lysis, and neutrophils were pelleted and resuspended in HBSS without divalent cations. Neutrophil purity was >95% in all experiments, and for EV isolation, neutrophils were resuspended at 20x10^6^ cells/mL in RPMI 1640 without phenol red. To obtain macrophages, buffy coat peripheral blood mononuclear cells were washed twice in Lonza RPMI 1640, resuspended at 2x10^6^ cells/mL in Lonza RPMI 1640 with 20% autologous serum (complete media), and incubated in Teflon jars at 37°C and 5% CO_2_ for 5-8 days. Cells were then washed, resuspended at 5x10^5^ cells/mL in Lonza RPMI 1640 with 10% pooled human serum, and plated in 6-, 12- or 24-well cell culture plates. Following at least two hours at 37°C and 5% CO_2_, cells were washed to remove lymphocytes and undifferentiated monocytes, and maintained in complete media.

### EV isolation

Neutrophils at 20x10^6^ cells/mL were left alone in buffer or treated with fMLF (1 µM), A23187 (4 µM), PMA (100 nM) or viable *S. aureus* at a 10:1 multiplicity of infection. Cells were incubated at 37°C for 20 minutes with inversion at 5-minute intervals, and EV production was halted by placing cells on ice for 10 minutes. Cells were then pelleted twice at 4000 x g for 20 minutes at 4°C, and supernatants containing EVs were collected. In some experiments, supernatants were treated with 0.05% TX-100 and vortexed for 30 seconds to lyse EVs (29), or filtered using a 0.2 µm polyethersulfone filter. EVs were pelleted by centrifugation at 160,000 x g for 51 minutes at 4°C, pooled, and pelleted again. EVs were resuspended in 200 µL PBS, aliquoted, flash-frozen, and stored at -80°C. EVs are referred to throughout by the agonist or treatment conditions used for their generation (e.g., A23187-EVs or TX-100-EVs). EV protein concentration was measured by bicinchoninic acid assay (BCA) based on manufacturer instructions.

### Cytotoxicity assay

Neutrophil and macrophage extracellular lactate dehydrogenase (LDH), an indicator of cell lysis, was measured in response to EVs, pharmacological agonists, and inhibitors using the Pierce cytotoxicity kit according to manufacturer instructions.

### Transmission electron microscopy

Negative-positive EV staining was performed as described in (30). Briefly, EVs were thawed and fixed with glutaraldehyde, before adding the EVs to formvar-coated copper grids by inversion of grids on EV droplets for 20 minutes. Following a series of washes, EVs were negative stained with methylcellulose-uranyl acetate, followed by positive staining with uranyl oxalate. Grids were left to dry for at least two hours before imaging using a Hitachi 7700 transmission electron microscope (Tokyo, Japan).

### Flow cytometry

EVs were conjugated to aldehyde/sulfate latex beads as described in (31). EVs were then stained with anti-CD63-PE for 30 minutes on ice. In separate experiments, macrophages were co-cultured with PKH67-stained A23187-EVs for the time periods, as indicated, harvested by trypsinization, and stained with anti-CD14-APC. In a subset of these experiments, macrophages were pre-treated with wortmannin or cytochalasin D as indicated elsewhere. All flow cytometry was performed on a Beckman Coulter CytoFLEX Flow Cytometer (Brea, CA).

### Immunoblotting

Cell lysates were prepared by resuspending primary human neutrophils at 20 x10^6^ cells/mL in 1x RIPA buffer with 1x Halt protease inhibitor cocktail and incubating on ice for 10 minutes, or by sonication as described in (28). Cell debris was pelleted at 18,400 x g for 10 minutes at 4°C, and supernatant was frozen at -80°C. Equal protein concentrations of EV subsets or cell lysates were combined with sample buffer, and boiled for 5 minutes at 95°C. Samples were resolved on a 4-15% SDS-PAGE gel at 120 V for 30 minutes to 1 hour. Proteins were transferred to PVDF membranes, blocked using 5% BSA or non-fat milk, then immunoblotted for GRP94, MPO, histone H3, citrullinated histone H3, TFAM, Bax, cytochrome c, GAPDH, or TOM20 at a 1:1000 dilution, and flotillin-1 and NE were used at a 1:20,000 dilution. All secondary antibodies were at a 1:20,000 dilution. Chemiluminescent substrate was used to visualize bands on a Bio-Rad ChemiDoc Touch imaging system (Hercules, CA).

### DNA quantification

Neutrophil extracellular DNA was measured using Sytox Green staining in a plate-based assay as described in (24, 32). EV surface DNA was quantified using the Quant-iT PicoGreen fluorescent staining kit based on manufacturer instructions. In other experiments, EV-DNA was isolated from 25–200 µL EVs using Qiagen’s DNeasy Blood and Tissue Kit. Following isolation, DNA was resolved on a 0.7% agarose gel stained with GelRed. For a subset of experiments, EVs were treated with and without DNase I prior to DNA isolation as described in (23).

### Droplet digital quantitative PCR

PCR reaction mixtures of 25 µL were prepared using 2.5 µL DNA, 1x Bio-Rad QX200 ddPCR EvaGreen Supermix, and 450 nM primers. A fragment of the chromosomal 18S was amplified with the forward primer: 5’-GTAACCCGTTGAACCCCATT-3’ and reverse primer: 5’-CCATCCAATCGGTAGTAGCG-3’ (33). Mitochondrial tRNA^Leu^ was amplified with forward primer: 5’-CACCCAAGAACAGGGTTTGT-3’ and reverse primer: 5’- TGGCCATGGGTATGTTGTTA -3’ (34). Resulting amplicons were quantified using the Bio-Rad QX200 Droplet Digital PCR System as previously described in (35). For each sequence, copy numbers were normalized to one million cell equivalents.

### Macrophage cytokine assays

EVs were thawed immediately prior to use. DNA was obtained from three sources: *E. coli* DNA was purchased commercially, whereas *S. aureus* DNA and EV-DNA were isolated using Qiagen’s DNeasy Blood and Tissue Kit and quantified by NanoDrop. For transfection, isolated DNA was combined with DOTAP at a 1:10 ratio for 15 minutes at room temperature. Macrophages were treated with EVs or DNA, and unless otherwise indicated, cells were incubated at 37°C and 5% CO_2_ for 18 hours. Supernatants were collected, pelleted at 18,400 x g for 10 minutes at 4°C, and flash frozen for later analysis by ELISA for IL-6 and IFN-α2, according to manufacturer instructions.

### Macrophage subcellular fractionation and analysis of mitochondrial membrane damage

Macrophages were subject to subcellular fractionation using digitonin as described in (36) with the following modifications: macrophages were resuspended at 5 x 10^5^ cells/mL for initial lysis in 200 µg/mL digitonin, 80 mM KCl in PBS, and 1x Halt protease inhibitor cocktail, and 1x RIPA buffer containing 1x Halt protease inhibitor cocktail was used as total cell lysis buffer. Fractions were then frozen at -80°C and analyzed by immunoblotting. In separate experiments, macrophages treated with or without spontaneous-EVs or A23187-EVs for 3-24 hours, and were harvested by trypsinization. Mitochondrial membrane depolarization was measured in these samples, along with carbonyl cyanide *m*-chlorophenyl hydrazone (CCCP)-treated macrophages, using the MitoProbe JC-1 assay per manufacturer instructions.

### Statistical analysis

Statistics were performed using Prism v9.3.1 (GraphPad, San Diego, CA, USA). Outliers were determined using the Grubb’s outlier test and excluded from analyses. For comparisons between multiple groups, one and two-way ANOVAs were performed. The statistical tests, *post-hoc* analyses, and *p*-values are indicated in figures and figure legends.

## Results

### Release of neutrophil-derived EVs during NETosis

**Figure 1:**
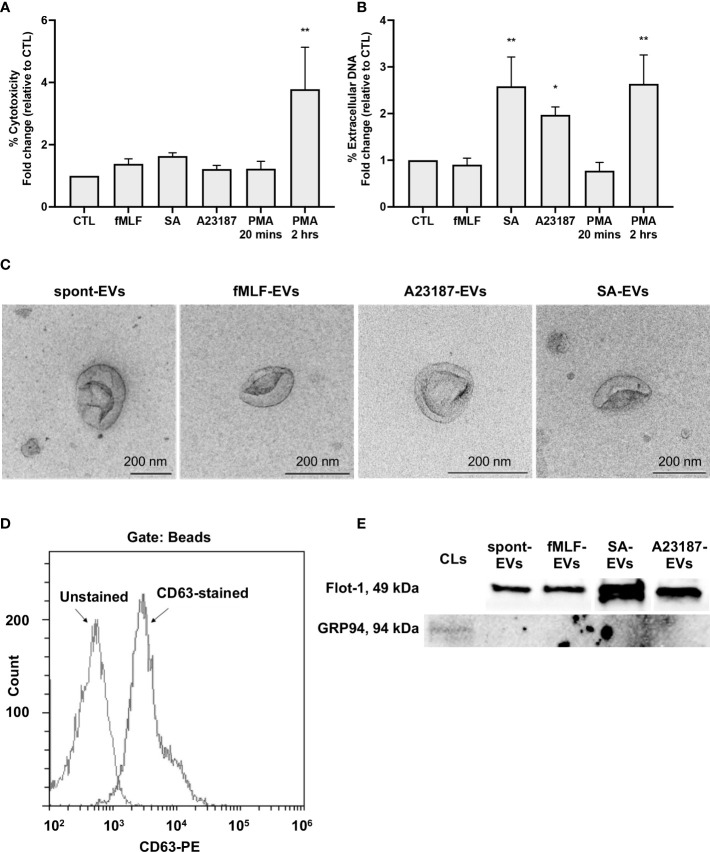
Neutrophils treated with NET agonists release EVs. Following 20 minutes incubation with buffer or indicated agonists, conditioned media was collected and assayed for cytotoxicity **(A**, n≥3**)** and extracellular DNA **(B**, n≥3**)**. Values are expressed as fold change relative to neutrophils left alone in buffer (control, CTL). Data are presented as the mean of three or more experiments ± SEM. *P*-values were determined using a one-way ANOVA with Dunnett’s post-test (* *p* < 0.05, ** *p* < 0.01 *vs.* CTL). EV subtypes (spontaneous-EVs (far left, scale bar 200 nm), fMLF-EVs (middle left, scale bar 200 nm), A23187-EVs (middle right, scale bar 200 nm), and *S. aureus*-EVs (far right, scale bar 200 nm)) were processed and then imaged on a Hitachi 7700 TEM at 80kV HR. Shown are representative electron micrographs at 10,000X magnification **(C**, n=3**)**. A23187-EVs were conjugated to beads, stained with anti-CD63-PE, and analyzed by flow cytometry. Shown is a representative histogram gated on bead-conjugated EVs **(D**, n=3**)**. Equivalent protein concentrations (1µg top and 9µg bottom) of cell lysates (CL) or EVs were separated by SDS-PAGE and blotted for flotillin-1 and GRP94. Shown are representative images of four or more experiments **(E)**.

First, to determine whether neutrophils treated with A23187 maintained their membrane integrity and released extracellular DNA, features of vital NETosis, we performed cytotoxicity and Sytox Green assays. Compared to control cells, neutrophils treated with fMLF, *S. aureus*, A23187, or PMA for 20 minutes did not release LDH into the supernatant (**Figure 1A**). Meanwhile, neutrophils treated with *S. aureus* or A23187 released DNA, consistent with vital NETosis (**Figure 1B**). Suicidal NETosis, a lytic form of NETosis, occurred with PMA treatment, but only following 2 hours. Using the pan-PAD inhibitor Cl-amidine, we attempted to establish whether citrullination of histone H3 by PAD4 was required for DNA release. Although PAD4 plays a role in some types of NETosis (37, 38), Cl-amidine failed to reduce NETosis in human neutrophils (25, 39), and did not block extracellular DNA release from neutrophils in response to A23187 (**Supplemental Figure 1A**).

Next, we isolated EVs when neutrophils were left alone in buffer, treated with fMLF, or undergoing vital NETosis in response to A23187 or *S. aureus*. In accordance with MISEV-2018 guidelines (40), we performed TEM, measured EV-enriched proteins CD63 and flotillin-1, and confirmed the absence of endoplasmic reticulum (ER) protein GRP94 to establish that material isolated by differential centrifugation represented EVs. Consistent with other reports (23, 30), EVs displayed cup-shaped morphology, and the majority of EVs had a diameter of less than 200 nm (**Figure 1C**). Further characterization by complementary analytical techniques confirmed that EVs possessed CD63 (**Figure 1D**) and flotillin-1, but not GRP94 (**Figure 1E**). These data confirm the recovery of neutrophil-derived EVs by differential centrifugation.

### A23187-EVs associated with NET proteins and DNA

**Figure 2:**
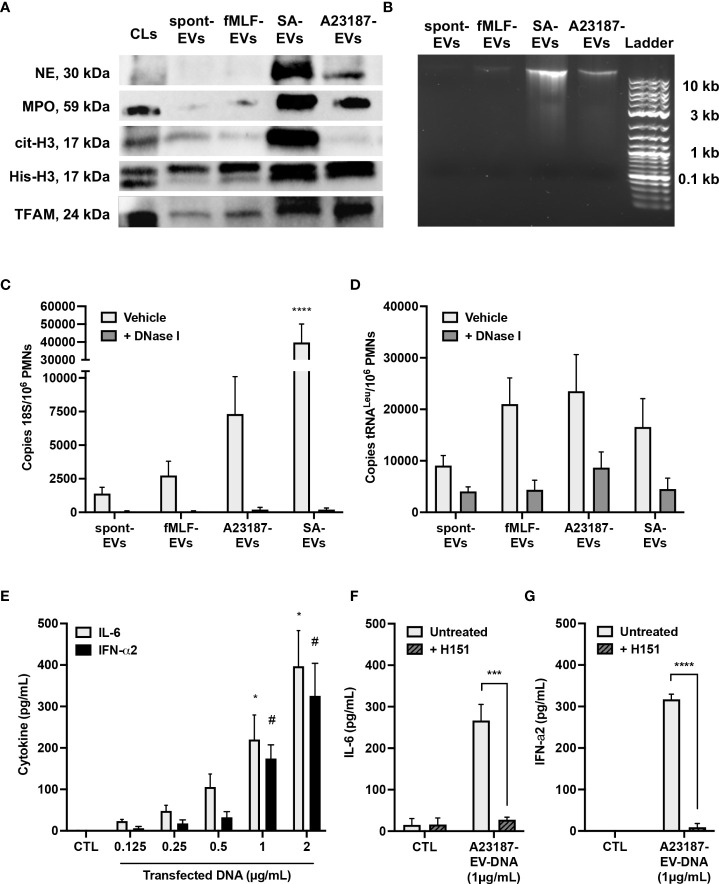
A23187-EVs associated with NET proteins and DNA, and transfected DNA activated cGAS-STING. Neutrophil elastase (NE), myeloperoxidase (MPO), citrullinated histone H3 (cit-H3), histone H3 (His-H3), and TFAM were detected by immunoblotting. Equivalent protein concentrations of cell lysates (CL) and EVs were separated by SDS-PAGE prior to immunoblotting (1µg for MPO; 3µg for NE, cit-H3, and His-H3; 9µg for TFAM). Shown are representative images of four experiments **(A)**. EV-DNA was isolated from EV subsets and resolved with gel electrophoresis. Shown is a representative image of five experiments **(B)**. EVs were treated or without with DNase I for 1 hour prior to isolating DNA. Primers specific for 18S rRNA and tRNA^Leu^ were used to amplify nuclear **(C)** and mitochondrial **(D)** DNA, respectively, and DNA copy numbers were quantified by ddPCR. Values are expressed as DNA copies per EV-cell equivalents. Bars represent the average of three or more experiments ± SEM. *P-*values were determined using a two-way ANOVA with Dunnett’s post-test (**** *p* < 0.0001 *vs.* spontaneous-EVs). A23187-EV-DNA was transfected for 18 hours at the indicated concentrations, and cytokines in supernatants were measured by ELISA **(E**, n=3**)**. Bars represent the average of experimental data ± SEM. *P* values were determined using a two-way ANOVA with Sidak’s post-test (* *p <*0.05 *vs.* CTL, ^#^ *p <*0.05 *vs.* CTL). EV-DNA was isolated from A23187-EVs and then encapsulated in DOTAP. Macrophages were pre-treated with and without H151 (1 µM), then transfected for 18 hours. Supernatants were collected and analyzed by ELISA for IL-6 **(F**, n=4**)** and IFN-α2 **(G**, n=3**)**. Bars represent the average of experimental data ± SEM. *P* values were determined using a two-way ANOVA with Sidak’s post-test (*** *p* < 0.001, **** *p* < 0.0001).

We next questioned whether components of NETs, such as granules proteins, DNA binding proteins, and DNA, attached to the isolated A23187-EVs. Neutrophil elastase and MPO were present in cell lysates, but lower levels of both proteins were associated with spontaneous and fMLF-EVs (**Figure 2A**). EVs isolated during NETosis, A23187-EVs and *S. aureus*-EVs, had greater amounts of neutrophil elastase and MPO compared to cell lysates, consistent with degranulation or NETosis. Next, we attempted to detect nuclear DNA-binding protein histone H3 (citrullinated and total), and mtDNA-binding protein TFAM, in EV subsets. Only *S. aureus*-EVs associated with citrullinated histone H3, but spontaneous-EVs, fMLF-EVs, and A23187-EVs did not. Although we could not detect elevated citrullinated histone H3 in A23187-EVs, A23187-EVs and *S. aureus*-EVs possessed more histone H3 and TFAM compared to spontaneous-EVs and fMLF-EVs, confirming the presence of NET associated proteins on EVs produced during NETosis.

Since we detected granule and DNA-associated proteins on A23187-EVs, we next measured and quantified EV-associated DNA. DNA isolated from spontaneous-EVs and fMLF-EVs resolved as an extremely faint band above 10 Kb (**Figure 2B**). However, a more pronounced band was apparent when DNA was isolated from *S. aureus*-EVs and A23187-EVs, albeit the A23187-EVs had qualitatively less DNA compared to *S. aureus*-EVs.

To more accurately quantify these differences, we isolated EV-DNA from each EV subset and performed droplet digital PCR (ddPCR) for nuclear and mitochondrial DNA sequences. Copies of 18S nuclear gene were present in DNA isolated from spontaneous-EVs, however copy number increased 2.0-fold in DNA isolated from fMLF-EVs, 5.3-fold for DNA isolated from A23187-EVs and 28.6-fold in DNA isolated from *S. aureus*-EVs (**Figure 2C**). Despite this trend, DNA copy number on A23187-EVs was highly variable, and therefore not significantly different from spontaneous-EVs. Treatment of EVs with the membrane impermeable endonuclease DNase I dramatically reduced copies of the 18S gene in all instances, showing that the majority of nuclear DNA was on the surface. MtDNA was measured by quantifying copies of tRNA^Leu^, a DNA sequence found in the mitochondrial genome. Compared to 18S copy number, tRNA^Leu^ copy number was 6.5-fold higher on spontaneous-EVs, suggesting that more mtDNA is constitutively released from neutrophils. Although tRNA^Leu^ copy number was higher in DNA isolated from fMLF-EVs, A23187-EVs, and *S. aureus*-EVs, these differences were not significantly different compared to spontaneous-EVs (**Figure 2D**). Following DNase I treatment, we found a greater percentage of DNase-resistant mtDNA, suggesting that a proportion of mtDNA resides within the EV lumen, but this trend did not correlate with NETosis.

### Transfected EV-DNA activated cGAS-STING

Given that A23187-EVs associate with nuclear and mtDNA, we explored the involvement of macrophage STING signaling in pro-inflammatory cytokine production driven by transfected DNA from A23187-EVs. To begin, we sought to determine the threshold for cytokine production following DNA transfection. We transfected A23187-EV-DNA and measured macrophage production of IL-6 and IFN-α2, products of the transcription factors NF-κB and IRF3, respectively. Compared to control cells, treatment with transfected DNA produced a dose-dependent increase in macrophage IL-6 and IFN-α2 (**Figure 2E**). We found that at least 1 µg/mL of DNA was needed to drive a significant increase in production of either cytokine, suggesting that lower concentrations of DNA (<1 µg/mL) may not activate macrophages. Since transfected NET DNA activates cGAS-STING (22), we tested whether EV-DNA was pro-inflammatory when delivered by DOTAP transfection. Macrophages transfected with DNA from A23187-EVs produced more IL-6, which decreased to baseline levels when cells were pre-treated with H151 (**Figure 2F**). Likewise, transfected macrophages produced increased IFN-α2 compared to untreated cells, which decreased to baseline levels when treated with H151 prior to transfection (**Figure 2G**).

### A23187-EVs are engulfed by macrophages in an actin- and PI3K-dependent manner

**Figure 3:**
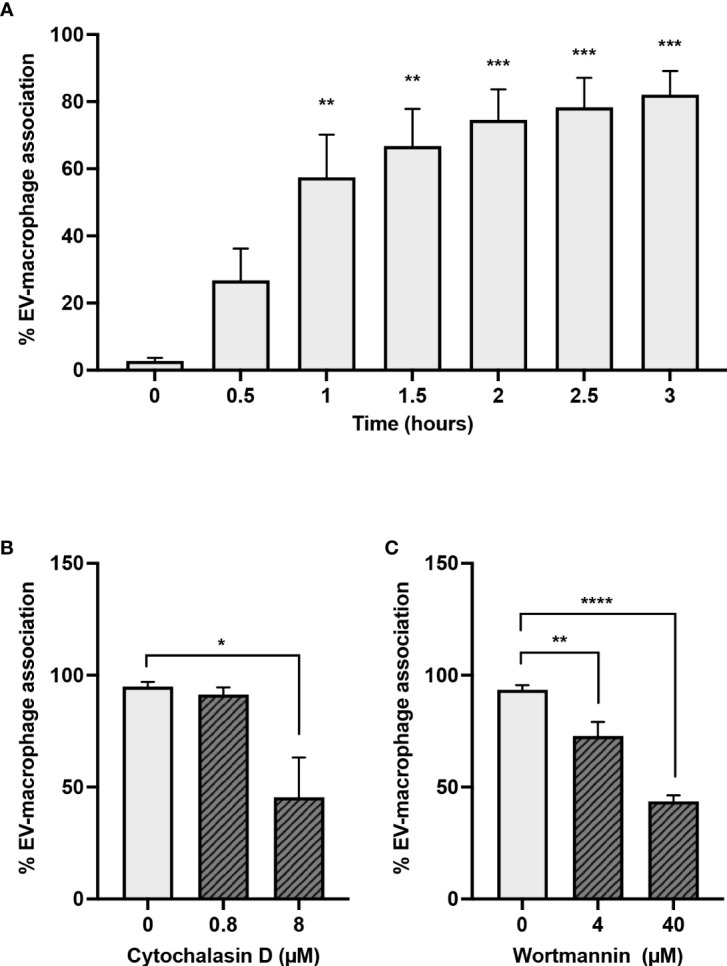
Macrophages engulf A23187-EVs in an actin- and PI3K-dependent manner. PKH67-labelled A23187-EVs were co-cultured with macrophages for the indicated times before analysis by flow cytometry **(A)**. Bars represent the average of three experiments ± SEM. *P* values were determined using a one-way ANOVA with Dunnett’s post-test (** *p* < 0.01, *** *p* < 0.001 *vs.* time 0). Macrophages were pre-treated for 2 hours with the inhibitors cytochalasin D **(B)** and wortmannin **(C)** at the indicated concentrations. PKH67-stained A23187-EVs were co-cultured with macrophages 1.5 hours, and analyzed by flow cytometry. Bars represent the average of three experiments ± SEM. *P* values were determined using a one-way ANOVA with Dunnett’s post-test (* *p* < 0.05, ** *p* < 0.01, **** *p* < 0.0001).

To investigate whether EVs produced during NETosis were pro-inflammatory during sterile inflammation, either because of associated DNA or other EV-associated cargo, we elected to use A23187-EVs, and determined whether macrophages would engulf them. We treated macrophages with A23187-EVs from 0.5-3 hours, and measured uptake by flow cytometry (**Figure 3A**). EV uptake was time-dependent, but after 1 - 1.5 hours, the rate of EV uptake had slowed, and at 3 hours 82.1% of CD14^+^macrophages had engulfed PKH67^+^EVs. To establish whether A23187-EV internalization was driven by an active process, such as endocytosis or macropinocytosis, we inhibited actin with cytochalasin D and phosphoinositide 3-kinase (PI3K) with wortmannin. Compared to untreated macrophages, treatment 0.8 µM cytochalasin D did not decrease macrophage internalization of PKH67^+^EVs; however, 8 µM cytochalasin D decreased EV uptake by 2.2-fold compared to untreated macrophages (**Figure 3B**). Similarly, at both concentrations tested, wortmannin-treated macrophages internalized less PKH67^+^EVs compared to untreated macrophages (**Figure 3C**).

### A23187-EVs cause macrophage IL-6 production

**Figure 4:**
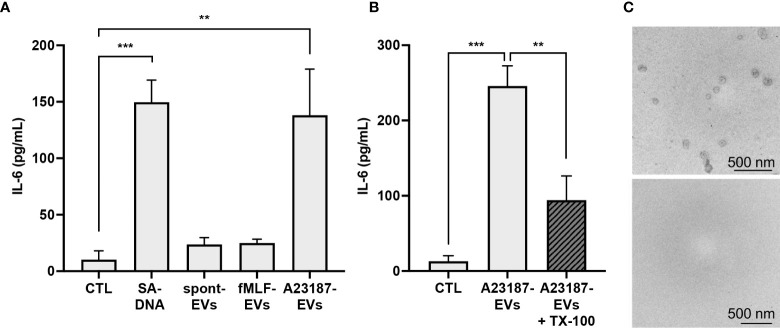
A23187-EVs cause macrophage IL-6 production. Macrophages were treated for 18 hours with 1 µg/mL of transfected *S. aureus* DNA (SA-DNA) or 1 µg/mL EV subtypes and supernatants were analyzed by ELISA **(A)**. A portion of A23187-EVs were lysed prior to ultracentrifugation with 0.05% TX-100, and paired whole and lysed A23187-EVs were used to treat macrophages at equivalent protein concentration as above **(B)**. Bars represent the averages of four or more experiments ± SEM. *P* values were determined using one-way ANOVA with Tukey’s post-test (** *p* < 0.01, *** *p* < 0.001). A23187-EVs treated without (top panel) and with (bottom panel) TX-100 were processed and imaged on a Hitachi 7700 TEM at 80kV HR. Shown are representative electron micrographs at 10,000X magnification **(C**, scale bar 500 nm, n=3**)**.

After establishing that A23187-EVs were engulfed by macrophages, we compared IL-6 production following macrophage treatment with A23187-EVs, to treatment with spontaneous-EVs, fMLF-EVs, and transfected DNA. Compared to control cells, macrophages produced 15-fold more IL-6 in response to DOTAP-transfected DNA, whereas neither spontaneous-EVs nor fMLF-EVs triggered IL-6 production (**Figure 4A**). Conversely, when compared to control macrophages, A23187-EVs induced 13.8-fold more IL-6 production. The A23187-EV-dependent increase in IL-6 production was diminished by 2.6-fold when A23187-EVs were lysed with the detergent TX-100 prior to isolation (**Figure 4B**), and lysis was confirmed by electron microscopy (**Figure 4C**), suggesting that intact A23187-EVs mediate the majority of this response.

### A23187-EVs cause cGAS-STING-dependent IL-6 production, but not IFN-α2

**Figure 5:**
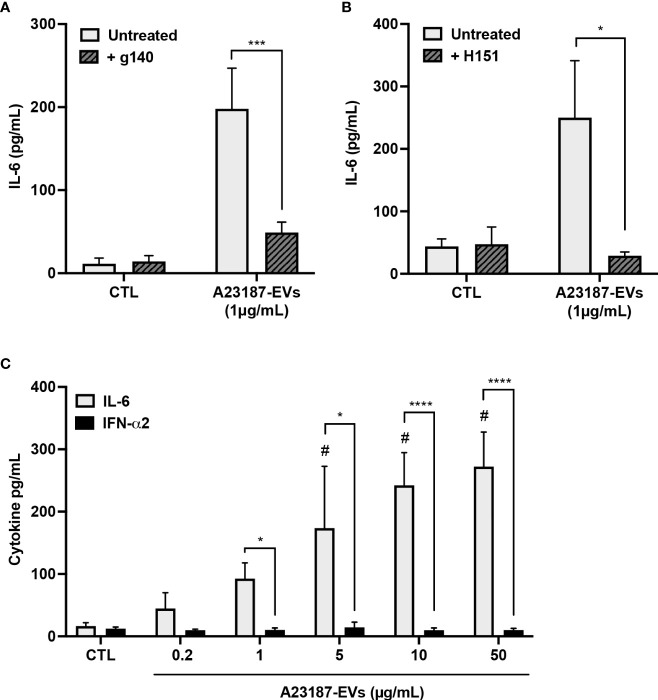
A23187-EVs cause cGAS-STING-dependent IL-6 production but do not cause IFN-α2 production. Macrophages were left in buffer or pre-treated with the inhibitors g140 **(A**, cGAS inhibitor, 5µM**)** or H151 **(B**, STING inhibitor, 1µM**)**. Macrophages were treated with A23187-EVs at the indicated concentrations, supernatants were collected after 18 hours, and IL-6 analysis was performed by ELISA. Bars represent the average of seven or more experiments ± SEM. *P* values were determined using a two-way ANOVA with Sidak’s post-test (* *p* < 0.05, *** *p* < 0.001 *vs.* CTL). Macrophages were treated with the indicated concentrations of A23187-EVs and cytokines were measured **(C**, n=11**)**. Bars represent the average of experimental data ± SEM. *P* values were determined using a two-way ANOVA with Sidak’s post-test (* *p <*0.05, **** *p <*0.0001; ^#^ *p* < 0.05 *vs.* CTL).

Since transfected EV-DNA activated STING, and A23187-EVs caused macrophage IL-6 production, we investigated whether A23187-EVs activated macrophage cGAS-STING signaling. To assess cGAS involvement and to exclude the possibility that EVs carried STING activator 2’3’ cGAMP as cargo, macrophages were pre-treated with or without cGAS small molecule inhibitor g140. A23187-EVs caused elevated IL-6 production, and this was significantly reduced to baseline levels when cells were pre-treated with g140 (**Figure 5A**). H151, the STING inhibitor, showed a similar pattern, where A23187-EVs caused on average 250 pg/mL IL-6 production, which was significantly reduced to baseline levels by pre-treatment with H151 (**Figure 5B**). Although there was also no macrophage cytotoxicity in the presence of either inhibitor (data not shown), we considered the possibility that g140 and H151 had off-target effects. To test for this, we exposed resting macrophages and inhibitor-treated macrophages to Toll-like receptor 3 agonist poly(I:C). Poly(I:C) treatment of macrophages resulted in IL-6 production, and as expected, neither g140 nor H151 diminished the elevated IL-6 levels (**Supplemental Figures 1B**, **C**), consistent with inhibitor specificity against cGAS and STING.

Canonical cGAS-STING activation also leads to production of type I interferons, so next we assayed the production of IFN-α2 by macrophages in response to A23187-EVs. Compared to control cells, treatment with A23187-EVs produced a dose-dependent increase in macrophage production of IL-6 (**Figure 2E**). Unexpectedly, macrophages treated with equivalent concentrations of A23187-EVs produced no increase in IFN-α2 (**Figure 5C**). We quantified DNA found in 50 µg/mL of A23187-EVs using the Quant-iT PicoGreen dsDNA assay. Less than 1 µg/mL of surface DNA was measured (0.81 µg/mL, data not shown), however unlike the pattern for transfected DNA (**Figure 2H**), IL-6 and IFN-α2 were uncoupled, and 1-50 µg/mL of A23187-EVs were still sufficient to cause elevated IL-6 production.

### A23187-EVs cause macrophage mitochondrial membrane depolarization, leading to cGAS-STING-dependent IL-6 production

**Figure 6:**
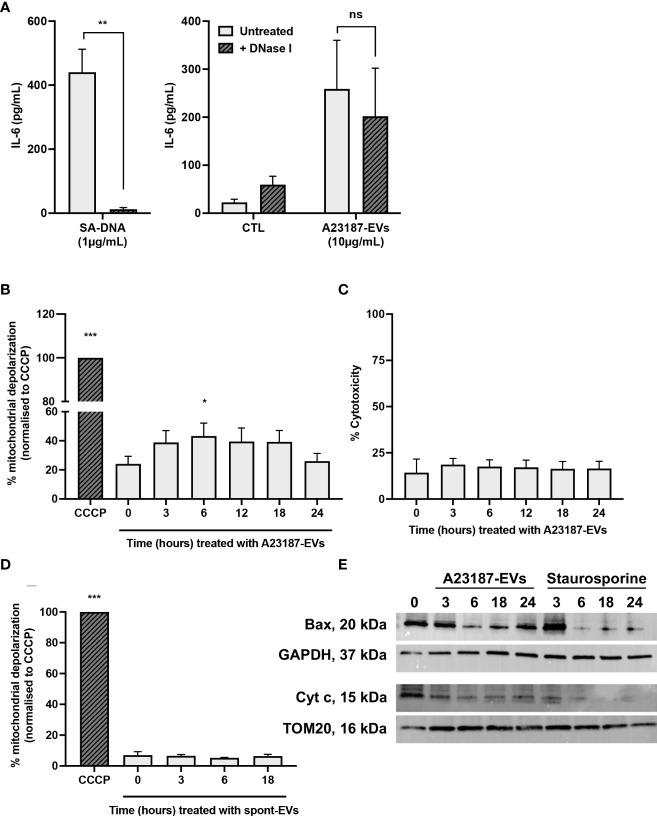
A23187-EVs cause macrophage mitochondrial dysfunction. *S. aureus* DNA (SA-DNA, left panel) and A23187-EVs (right panel) were treated with and without DNase I, then co-cultured with macrophages for 18 hours. Supernatants were analyzed by ELISA for IL-6 **(A)**. Bars represent the average of 8 experiments ± SEM. *P* values were determined using a paired t-test (left panel, ** *p* < 0.01) and a two-way ANOVA with Sidak’s post-test (right panel, ns, not significant,). Macrophages were left untreated, or were treated at the indicated times with A23187-EVs or CCCP, stained using JC-1, then analyzed by flow cytometry for loss of mitochondrial membrane polarization **(B)**. Data was normalized to CCCP treatment and bars represent the average of 5 experiments ± SEM. *P* values were determined using a one-way repeated measures ANOVA with Dunnett’s post-test (* *p* < 0.05, *** *p* < 0.001 *vs.* time 0). Following treatment of macrophages with A23187-EVs for the indicated time points, supernatants were collected and assayed LDH as a measure for cytotoxicity **(C**, n=3**)**. Values are expressed as fold change relative to untreated macrophages. Data are presented as the mean of 3 experiments ± SEM. Macrophages were treated at the indicated times with spontaneous-EVs or CCCP, then analyzed by flow cytometry as above **(D)**. Bars represent the average of three experiments ± SEM. *P* values were determined using a one-way repeated measures ANOVA with Dunnett’s post-test (* *p* < 0.05, *** *p* < 0.001 *vs.* time 0). Macrophages were treated with A23187-EVs at the indicated time points, subcellular fractionation performed, then 10 µg lysates were resolved by SDS-PAGE gel and blotted for Bax, cytochrome c, and loading controls GAPDH and TOM20, as indicated **(E)**. A representative result from four experiments is shown.

To begin to resolve the mechanism underlying cGAS-STING dependent IL-6 production in response to A23187-EVs, we assessed whether surface DNA associated with A23187-EVs was driving cytokine production. First, we confirmed DNase-mediated digestion of DNA and assessed its impact on macrophage IL-6 production. Isolated DNA was treated with or without DNase I and transfected using DOTAP. In response to transfected DNA, macrophages produced 440.3 pg/mL of IL-6, and IL-6 production was abolished when DNA was degraded with DNase I (**Figure 6A**, left panel). Next, we treated macrophages with A23187-EVs or DNase-treated A23187-EVs and measured IL-6 production. In response to untreated A23187-EVs or DNase-treated EVs, macrophages produced comparable levels of IL-6 (**Figure 6A**, right panel). These data suggest that surface DNA does not play a role in cGAS-STING dependent IL-6 production in response to A23187-EVs.

Mitochondrial damage and release of mtDNA into the cytosol activated cGAS-STING dependent IL-6 production without type I interferon in a model of cisplatin-induced acute kidney injury (27). Given these results, we investigated the possibility that A23187-EVs caused leakage of mtDNA into the cytosol of macrophages. Loss of mitochondrial membrane potential due to the recruitment of the pore-forming protein Bax can facilitate escape of mtDNA (41, 42). We assessed macrophage mitochondrial membrane depolarization using a JC-1 assay. Cells were treated with CCCP, an agonist for membrane depolarization, and used as the basis for comparison to A23187-EV-treated macrophages. Compared to untreated cells, a 1.8-fold increase in mitochondrial membrane depolarization was observed following 6 hours of stimulation with A23187-EVs (**Figure 6B**). Depolarization returned to baseline by 24 hours, and no LDH release occurred during this time period (**Figure 6C**), suggesting that mitochondrial depolarization did not immediately lead to apo-necrosis. Consistent with membrane depolarization leading to IL-6 production in response to A23187-EVs, but not spontaneous-EVs, no mitochondrial membrane depolarization was observed when macrophages were treated with spontaneous-EVs for the same time period (**Figure 6D**).

Mitochondrial membrane depolarization is consistent with MOMP. During MOMP, Bax is depleted from the cytosol and forms pores in the mitochondrial membrane (43, 44). Once the pore is formed, cytochrome c exits the inner mitochondrial membrane space and appears in the cytosol. We performed subcellular fractionation of cytosol and mitochondrial membranes following A23187-EV treatment of macrophages to quantify loss of cytosolic Bax and loss of mitochondrial cytochrome c. Bax was present in the cytosolic fraction of macrophages, and its levels decreased following treatment with A23187-EVs or staurosporine, a kinase inhibitor that induces apoptosis (**Figure 6E**). Interestingly, Bax levels began to increase by 24 hours in macrophages treated with A23187-EVs, but not staurosporine-treated macrophages. Corresponding to a decrease in cytosolic Bax, cytochrome c in the mitochondrial fraction declined over time in macrophages treated with either A23187-EVs or staurosporine, suggesting that mitochondrial damage occurs in macrophages treated with A23187-EVs.

## Discussion

Neutrophil-derived EVs and NETs are elevated in sepsis and other inflammatory conditions, and questions remain as to whether EVs generated concurrently with NETosis are pro-inflammatory. Here, we showed that EVs generated from neutrophils treated with A23187 cause cGAS-STING-dependent pro-inflammatory IL-6 production by primary human macrophages. A23187-EVs and associated DNA were characterized, and EV-DNA transfected into macrophages caused cGAS-STING-dependent IL-6 and type I interferon production. By comparison, A23187-EVs only caused IL-6 production. This response occurred following DNase I-digestion of EV-DNA, suggesting that while EVs produced during NETosis are pro-inflammatory in macrophages, this is not dependent on surface-associated DNA. In response to A23187-EVs, macrophages exhibited increased mitochondrial dysfunction, suggesting that macrophage mtDNA may activate the cGAS-STING signaling axis. Overall, we identify an unexpected mechanism by which EVs produced by neutrophils during NETosis cause EV-driven activation of DNA-sensing pattern recognition receptors.

DNA associates with EVs isolated from multiple cell types (31, 45–47). Of the resulting EV subtypes analyzed, only those generated using *S. aureus* associated with significantly more surface nuclear DNA, highlighting that EVs generated in response to whole viable pathogens differ from chemical agents, including A23187. By gel electrophoresis, A23187-EVs associated with more DNA than fMLF-EVs or spontaneous-EVs; however, by ddPCR, this trend was not significant. This was not expected because A23187- and *S. aureus*-treated neutrophils released comparable amounts of DNA, and comparable levels of MPO, elastase, and histone H3 proteins were detected on A23187-EVs and *S. aureus*-EVs. EVs vary depending on agonists used (2, 3, 48, 49), and while additional studies are needed to characterize *S. aureus*-EVs and other EVs produced during infection, we continued to focus on A23187-EVs and their associated DNA, which lack bacteria and bacteria-associated molecules that might also activate pattern recognition receptors.

Similar to the response observed for transfected NETs (22, 50, 51), transfected DNA isolated from A23187-EVs caused STING-dependent IL-6 and type I interferon in primary human macrophages. In contrast, A23187-EVs caused cGAS-STING-dependent macrophage production of IL-6 without type I interferon, suggesting that in this instance, activation of NF-κB occurs independently of IRF-3-driven type I interferon production. Macrophages pre-treated with A23187-EVs produced type I interferon when subsequently transfected with DNA (data not shown), suggesting that type I interferon production was not actively inhibited, but that non-canonical STING activation leading only to NF-κB activation occurred. The mechanism for disabling interferon synthesis remains elusive, but could involve defects in STING trafficking and post-translational modifications (52–54). Regardless, this system allows for further exploration of STING-mediated NF-κB signaling. Type I interferon-independent activities of STING have been discovered in recent years (reviewed in 55), and the co-culture of cells with A23187-EVs may help better define the role of STING-induced NF-κB in infection, cancer, and autoimmune diseases.

Nuclear and mtDNA, either on or within some EVs can activate DNA-sensing pattern recognition receptor pathways (56–58). However, we uncovered an unexpected mechanism for pro-inflammatory cytokine production driven by A23187-EVs. After rejecting the hypothesis that surface DNA resulted in cGAS activation, we considered the possibility that macrophage mtDNA was responsible for IL-6 production. A study by Maekawa and colleagues showed that mitochondrial damage and subsequent mtDNA leakage into the cytosol through mitochondrial Bax pores caused non-canonical cGAS-STING activation (27, 41). After treatment of macrophages with A23187-EVs, we observed mitochondrial membrane depolarization, along with decreased mitochondrial cytochrome c and decreased cytosolic Bax, suggesting MOMP occurred. The signals leading to mitochondrial damage are currently under investigation; however, prior work showed that neutrophil granule proteins including neutrophil elastase (59) and MPO (26), as well as oxidized phospholipids (60), induce a stress response in macrophages.

In summary, we have shown that treatment of macrophages with neutrophil-derived EVs generated using A23187 causes non-canonical cGAS-STING-dependent IL-6 production. We demonstrated that this increase was not driven by macrophage sensing of DNA on A23187-EVs. Instead, we found that A23187-EVs increased macrophage mitochondrial membrane depolarization, and corresponding decreases in cytosolic Bax and mitochondrial cytochrome c were observed. Characterization of EVs produced during NETosis will provide insight into inflammatory conditions where these neutrophil products are elevated. Additionally, A23187-EVs may be employed to study and treat STING-mediated inflammatory diseases, including infection, cancer, and autoimmunity.

## Data availability statement

The original contributions presented in the study are included in the article/**supplementary material**. Further inquiries can be directed to the corresponding author.

## Ethics statement

The studies involving human participants were reviewed and approved by Institutional Review Board, Central Michigan University. The patients/participants provided their written informed consent to participate in this study.

## Author contributions

EA contributed to study design, execution of experiments, data analysis, and writing the manuscript. KW-K contributed to execution of experiments, data analysis, and editing of the manuscript. EP contributed to execution of the experiments, data analysis, and editing of the manuscript. AM contributed to planning of experiments and editing of the manuscript. MG-W conceptualized and funded this study, and contributed to data analysis and writing the manuscript. All authors contributed to the article and approved the submitted version.

## Funding

This work was supported by the National Institute of General Medical Sciences of National Institutions of Health for award number R15GM132992, in addition to start-up funds from Central Michigan University. The content is solely the responsibility of the authors and does not necessarily represent the official views of the National Institutes of Health.

## Acknowledgments

The authors thank Hunter Galvin, Mary-Catherine Reeves, Danielle Mason, and Maia Fleck (Central Michigan University) for their technical assistance.

## Conflict of interest

The authors declare that the research was conducted in the absence of any commercial or financial relationships that could be construed as a potential conflict of interest.

## Publisher’s note

All claims expressed in this article are solely those of the authors and do not necessarily represent those of their affiliated organizations, or those of the publisher, the editors and the reviewers. Any product that may be evaluated in this article, or claim that may be made by its manufacturer, is not guaranteed or endorsed by the publisher.
