# Supplementary figures and images for: Extracellular vesicles from A23187-treated neutrophils cause cGAS-STING-dependent IL-6 production by macrophages
Source: Front Immunol. 2022 Jul 29;13:949451. doi: 10.3389/fimmu.2022.949451 (PMC9374307; doi:10.3389/fimmu.2022.949451)

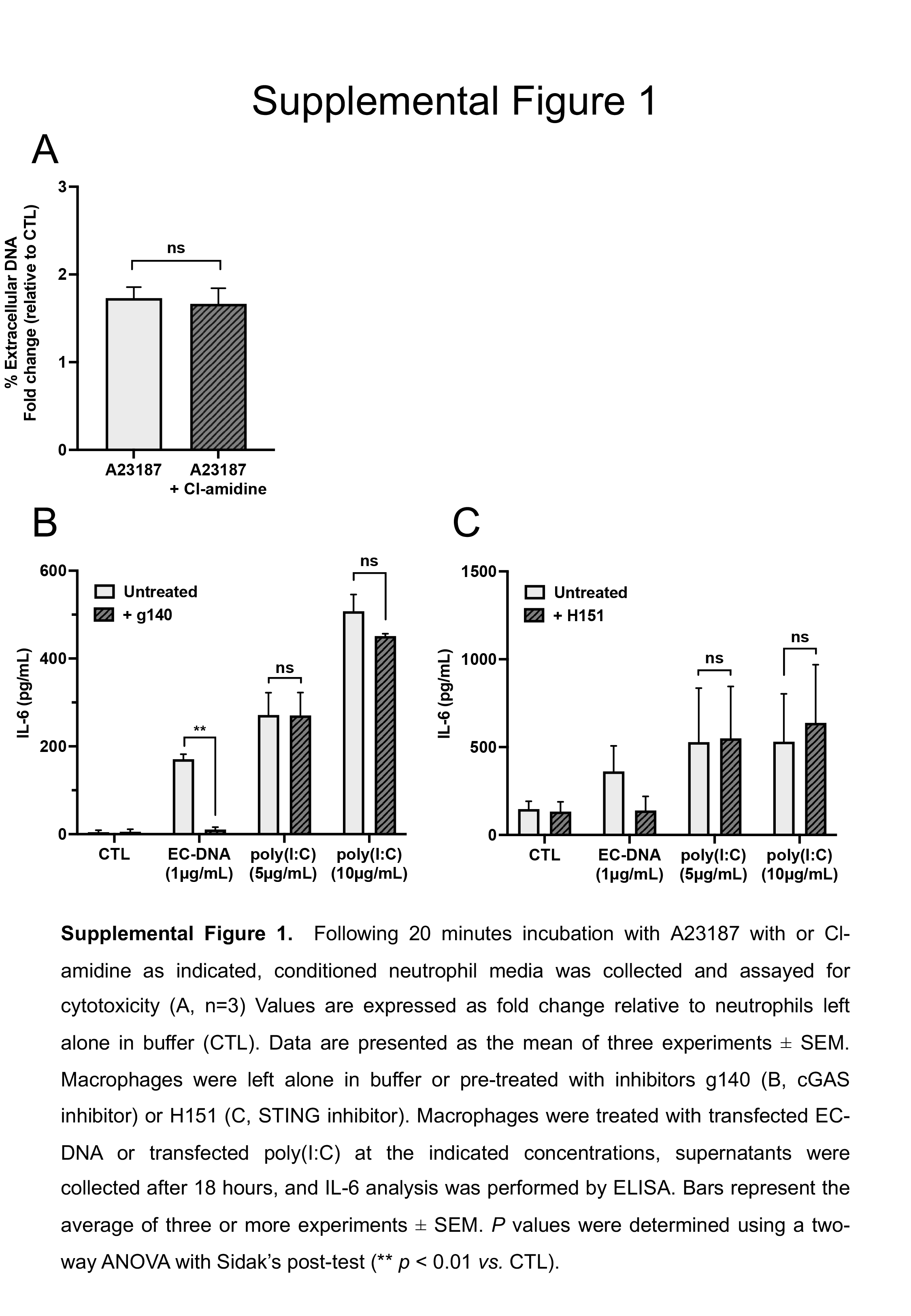

Supplement: Supplementary file 1 [file Image_1.tif]
